# Epilepsy disease classification: a community effort to enhance the Mondo Disease Ontology

**DOI:** 10.1093/database/baag004

**Published:** 2026-02-16

**Authors:** Nicole Vasilevsky, Sarah Gehrke, Kathleen Mullen, Subit Barua, Ian Braun, Tobias Brünger, Curtis Coughlin, Alina Ivaniuk, Daniel Korn, Dennis Lal, Stephanie Marsh, Elaine O’Loughlin, Daniel Olson, Yousif Shwetar, Christalena Sofocleous, Vanessa Vogel-Farley, Heidi Grabenstatter, Melissa Haendel, Christopher Mungall, Sabrina Toro

**Affiliations:** Critical Path Institute, 1840 E River Rd, Suite 100 Tucson, AZ 85718-5960, United States; The University of North Carolina at Chapel Hill, 120 Mason Farm Road, 5000 D Genetic Medicine Building CB#7264, Chapel Hill, NC 27599-7264, United States; The University of North Carolina at Chapel Hill, 120 Mason Farm Road, 5000 D Genetic Medicine Building CB#7264, Chapel Hill, NC 27599-7264, United States; Department of Pathology, Anatomy & Laboratory Medicine, West Virginia University, Health Sciences Campus, 763 Chestnut Ridge Rd. Morgantown, WV 26505, United States; Critical Path Institute, 1840 E River Rd, Suite 100 Tucson, AZ 85718-5960, United States; The University of Texas Health Science Center at Houston, 7000 Fannin, Suite 1706. Houston, Texas 77030, United States; University of Colorado Anschutz, 13001 E 17th Pl, Aurora, CO 80045, United States; Mayo Clinic Florida, 4500 San Pablo Road Jacksonville, FL 32224, United States; The University of North Carolina at Chapel Hill, 120 Mason Farm Road, 5000 D Genetic Medicine Building CB#7264, Chapel Hill, NC 27599-7264, United States; The University of Texas Health Science Center at Houston, 7000 Fannin, Suite 1706. Houston, Texas 77030, United States; Critical Path Institute, 1840 E River Rd, Suite 100 Tucson, AZ 85718-5960, United States; CURE Epilepsy, 420 N. Wabash Avenue, Suite 650. Chicago, IL 60611, United States; Critical Path Institute, 1840 E River Rd, Suite 100 Tucson, AZ 85718-5960, United States; The University of North Carolina at Chapel Hill, 120 Mason Farm Road, 5000 D Genetic Medicine Building CB#7264, Chapel Hill, NC 27599-7264, United States; National and Kapodistrian University of Athens School of Medicine, 75 Mikras Asias Street, Goudi, 11527 Athens, Greece; Rare Epilepsy Network, 12042 Cedar Creek Rd, Cedarburg, WI 53012, United States; Critical Path Institute, 1840 E River Rd, Suite 100 Tucson, AZ 85718-5960, United States; The University of North Carolina at Chapel Hill, 120 Mason Farm Road, 5000 D Genetic Medicine Building CB#7264, Chapel Hill, NC 27599-7264, United States; Genomics, Lawrence Berkeley National Laboratory, 1 Cyclotron Road, Berkeley, CA 94720, United States; The University of North Carolina at Chapel Hill, 120 Mason Farm Road, 5000 D Genetic Medicine Building CB#7264, Chapel Hill, NC 27599-7264, United States

## Abstract

Motivation: Epilepsy is a diverse group of neurological disorders affecting over 50 million people worldwide. While common epilepsy types are well studied, rare epilepsies—often severe and genetically complex—pose significant challenges in diagnosis, research, and treatment. Accurate and interoperable etiology and disease classifications are critical for improving data sharing, supporting clinical decision-making, and advancing rare disease research. Results: To enhance the accuracy of epilepsy-related disease concept representation within the Mondo Disease Ontology (Mondo), we conducted a series of expert-driven workshops in collaboration with the team from the Rare Disease Cures Accelerator-Data and Analytics Platform (RDCA-DAP). Specialists in epileptology, genetics, neurodevelopment, biomedical ontology, and patient community advocates systematically reviewed and revised the epilepsy hierarchy in Mondo, aligning it with the International League Against Epilepsy (ILAE) classification system. These updates include reclassification of epilepsy subtypes, including syndromes, age-related epilepsies, and developmental epileptic encephalopathies, resulting in a more granular, standardized, and clinically relevant structure. Mondo now offers an enhanced framework for integrating epilepsy data across resources, enabling improved interoperability and facilitating rare disease research and data curation, with continued efforts underway to further refine and expand this integration.

## Introduction

Epilepsy, a neurological disorder characterized by recurrent seizures due to abnormal electrical activity in the brain, affects millions globally and significantly impacts quality of life. Recent data indicates that ∼51.7 million individuals worldwide were living with epilepsy in 2021 [1], underscoring its global public health burden. While common generalized and focal epilepsies are better understood clinically, more complex and rare epilepsies—a subset of rare diseases which are characterized by affecting fewer than 1 in 2000 individuals [2]—pose distinct and complicated challenges. These include delayed diagnosis, limited treatment options, and a lack of robust clinical research [3]. Rare epilepsies, such as developmental and epileptic encephalopathies (DEEs), frequently present in infancy or early childhood and are often associated with intellectual disabilities, behavioural comorbidities, and drug-resistant seizures [4]. As a result, these conditions demand highly individualized, multidisciplinary approaches to diagnosis and care, including genetic testing, neurodevelopmental assessments, and targeted therapies [5].

Defining, standardizing, and unifying epilepsy disease concepts into a terminological classification system provides a structured, standardized framework for representing the complex and heterogeneous nature of epilepsy. By organizing disease concepts such as epilepsy subtypes, syndromes, and genetic epilepsies in a consistent and interoperable way, an ontology enhances data integration across research studies, supports clinical decision-making, and facilitates more precise diagnosis and personalized care, as well as providing an improved framework for future data collection. Ontologies provide structured definitions of the disease concepts, in both human-readable and machine-readable formats, that capture the defining characteristics of a disease such as causes, associated phenotypes, onset, progression, and classification. It also enables computational tools to more effectively analyse and share knowledge, driving advancements in both research and clinical practice.

There are various terminologies and ontologies that organize and standardize information about epilepsy, capturing multiple knowledge frameworks and classifying disease concepts in ways that reflect different perspectives (e.g. geneticists, epileptologists, physicians, and academics). There are several epilepsy domain-specific ontologies, which are used for various research and clinical care purposes. For example, the Epilepsy and Seizure Ontology [6] has been used in epilepsy-focused research within healthcare settings, such as in the Cloudwave platform—a neuroinformatics system designed to manage and analyse multimodal data (e.g. EEG, imaging, and clinical records) for patients with epilepsy [7]. A full list of available epilepsy-specific ontologies are listed in Table 1. Epilepsy-related disease concepts are represented from broader disease resources and ontologies, such as rare epilepsies in Orphanet [8], Mendelian seizure disorders in Online Mendelian Inheritance in Man (OMIM) [9], and Systematized Nomenclature of Medicine—Clinical Terms (SNOMED CT), which provides a structured representation of general epilepsy types, comorbidities, diagnostic procedures, and related clinical concepts [10]. While individually comprehensive, the variety of sources and application targets has led to significant overlaps and inconsistencies across the classifications.

**Table 1:** Existing epilepsy-specific disease terminologies.

Terminology name	Terminology abbreviation	Description
Epilepsy Ontology	EPIO	An Open Biological and Biomedical Ontology (OBO) Foundry ontology that aims to enhance understanding of epilepsy by organizing concepts related to classification, diagnosis, treatment, and disease mechanisms. EPIO integrates information from the ILAE classification, as well as other epilepsy-related ontologies, such as Epilepsy Syndrome Seizure Ontology (ESSO) and Epilepsy Semiology (EPISEM) [31].
Epilepsy Ontology	EPILONT	EPILONT is an ontology designed to support epilepsy research and classification. It is based on the diagnostic framework proposed by the ILAE and provides a formal representation of epilepsy-related concepts [32].
Epilepsy Syndrome Seizure Ontology-	ESSO	ESSO is designed to categorize epilepsy syndromes, seizure types, and associated data elements. It provides a framework for organizing epilepsy-related knowledge in a machine-readable format, making it useful for researchers and clinicians [33].
Epilepsy Semiology	EPISEM	EPISEM is designed to capture the semiology of epilepsy, including ictal, post-ictal, inter-ictal, and aura signs. It provides a structured framework for classifying and analysing epilepsy-related symptoms and their manifestations [34].
Epilepsy and Seizure Ontology	EPSO	An application ontology developed to support epilepsy research, patient care, and health system management. It integrates structured knowledge on epilepsy syndromes, seizure types, and neuropathology terms, including immunohistochemistry and anatomical descriptions. EPSO incorporates terminology from ILAE and the National Institute of Neurological Disorders and Stroke [6, 35].

**Figure 1:**
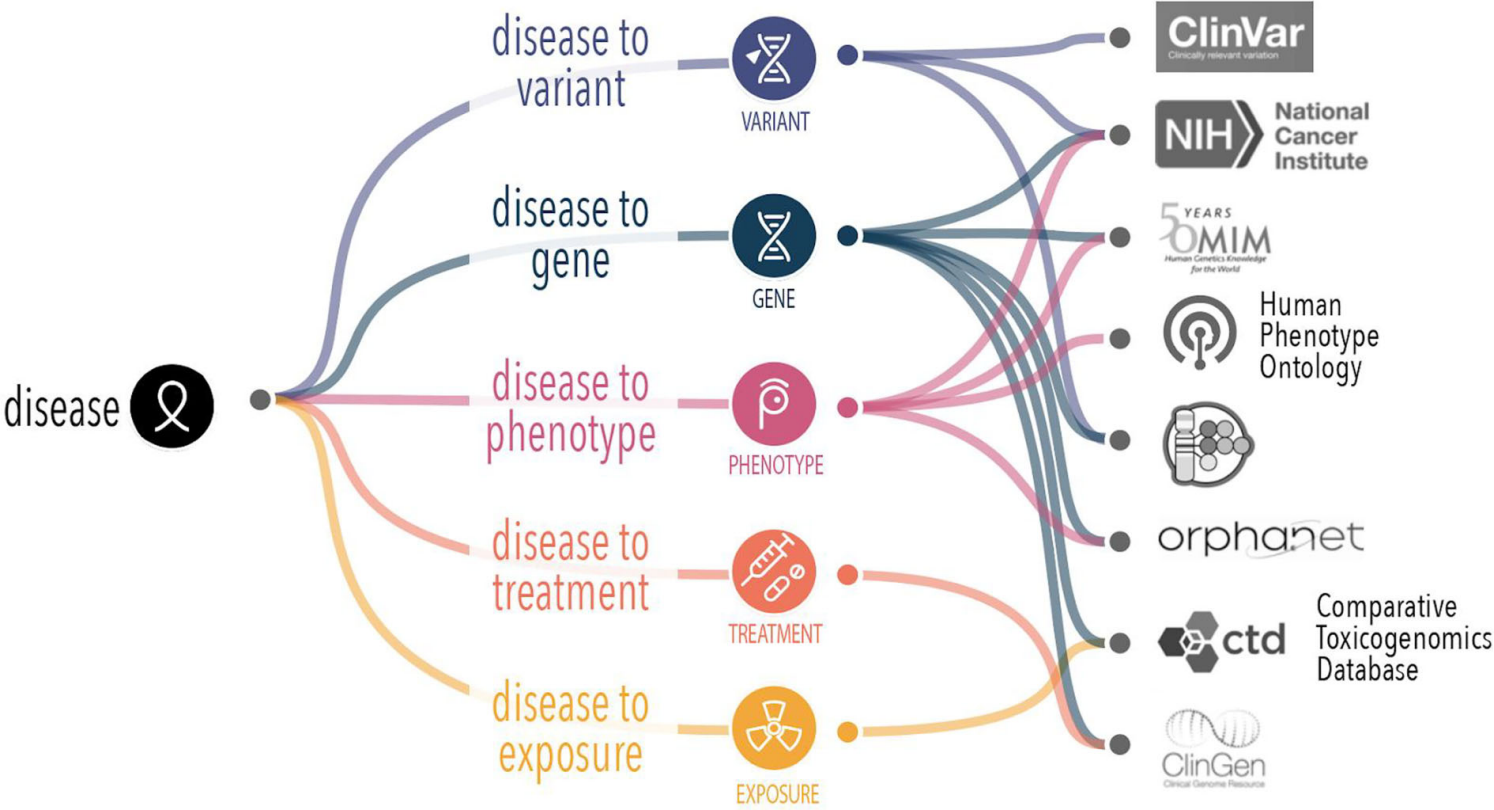
Mondo supports alignment of different disease attributes that are captured in different sources. In order to form a complete picture of knowledge about a given disease, we need an authoritative handle (stable reference) to robustly and reproducibly collate disease features.

The Mondo Disease Ontology (Mondo) bridges the gap between the otherwise disconnected disease concept and ontological systems. Mondo is used for disease annotations in multiple applications to facilitate accurate data sharing, research, and clinical applications across diverse healthcare systems. It offers a cross-species, comprehensive ontology that provides a standardized and consistent framework for classifying diseases, including an extensive classification of epilepsy and related seizure disorders, providing a structured, hierarchical system for organizing and defining epilepsy concepts [11]. Mondo integrates multiple disease classifications, such as Orphanet, OMIM, and the Human Disease Ontology [12] and others, offering a unified approach to understanding epilepsy within the broader context of neurological and systemic conditions (Fig. 1). This multi-sourced mapping supports interoperability between systems and aids in the diagnosis of rare and complex forms of epilepsy that may only have information in one database. While Mondo integrates many epilepsy-related concepts from a broad range of biomedical ontologies, it does not aim to exhaustively cross-reference every term from all external epilepsy-specific terminologies.

The representation and classification of epilepsy- and seizure-related disease concepts in Mondo are based on the integration of external terminologies and classification systems (listed here: https://mondo.monarchinitiative.org/pages/sources/). As new concepts and classifications emerge, it is essential to keep existing definitions current and aligned with the latest medical understanding.

The International League Against Epilepsy (ILAE) (https://www.epilepsydiagnosis.org/index.html) is the leading global organization dedicated to advancing the understanding, diagnosis, and treatment of epilepsy [13, 14]. The ILAE plays a central role in promoting research, education, and clinical care for people with epilepsy worldwide. It is best known for developing and regularly updating standardized classifications for seizure types and epilepsy syndromes, which are widely adopted in clinical and research settings. These classifications help ensure consistency in diagnosis and communication across healthcare systems. The ILAE also fosters international collaboration, supports training programmes, and advocates for policies that improve epilepsy care, especially in low- and middle-income countries. Through its efforts, the ILAE continues to shape the global epilepsy landscape and improve outcomes for individuals affected by the condition.

To improve the representation of epilepsy-related disease concepts in Mondo, a series of focused virtual workshops were conducted in collaboration with domain experts from diverse areas of epilepsy research and clinical practice to review the current representation of the Mondo epilepsy hierarchy and better align with the classification in the ILAE. Discussions also covered other clinically relevant classification approaches and related efforts in the field.

## Workshop approach and decision framework

The virtual workshops were held over three sessions in the fall and winter of 2024–2025 (designated Part 1, 2, or 3) for ∼2 h per session. This workshop series aimed to review, refine, and expand epilepsy classifications within Mondo, ensuring alignment with current medical knowledge and community standards. We followed the Mondo established process, including the consensus-building approach detailed in Vasilevsky *et al*. 2025 [11]. We convened community stakeholders and subject matter experts to systematically review the classification and representation of epilepsy disease entities in Mondo. Workshop participants included ontology experts, clinical experts, including specialists in epileptology and neurodevelopmental genetics, and rare disease patient advocates, some of whom had lived experience with complex epilepsy (Table 2).

**Table 2:** Summary of workshop attendees from the three-part epilepsy workshops.

Session number	Total attendees	Ontology experts	Clinical experts	Patient advocates
Part 1	**14**	7	5	2
Part 2	**14**	6	7	1
Part 3	**19**	7	10	2

The total number of attendees at each session are noted in the second column. The workshop participants (27 unique participants across the three sessions) consisted of ontology experts, clinical experts, and patient advocates.

We first identified key areas of focus and compiled them into a slide deck that guided discussion (available here: https://zenodo.org/records/17834824). Specific questions were prepared in advance and reviewed collectively by workshop participants. As Mondo is a community-owned resource and feedback is always welcome outside of the workshops, all of the discussions and decisions were recorded in our GitHub issue tracker and tagged with an ‘epilepsy’ label (https://github.com/monarch-initiative/mondo/issues?q=is%3Aissue%20state%3Aopen%20label%3Aepilepsy). For consensus building, we proposed revisions that were non-controversial (i.e. all the workshop participants were in agreement) were readily agreed upon and documented in GitHub tickets prior to implementation, allowing the broader community an opportunity to comment. When consensus could not be reached during the workshop, GitHub tickets were created to support continued asynchronous discussion. The Mondo community members were informed and invited to participate in the discussions and decisions (either during the workshops or asynchronously) via the Mondo monthly email, outreach calls, and material available on our website. It should be noted that some discussions did not lead to consensus. These are recorded in GitHub tickets that remained open and will be addressed as agreement is reached within the community. This approach, pairing subject-matter experts with ontology curators, is a well-established and effective method for Mondo development [11]. The combination of clinical domain expertise and the specialized knowledge of ontology developers enabled clinical insights to be translated into logically consistent, well-structured representations aligned with ontology best practices, ensuring that content was both accurate and semantically robust.

## Reclassification of Mondo epilepsy concepts

The primary focus was to review the epilepsy branch in Mondo (MONDO:0005027) and the current classification of epilepsy disease concepts in the ILAE that was released in 2022 [13] with the aim of identifying and resolving discrepancies.

A significant number of changes to the epilepsy classification were implemented based on the workshop discussions. It should be noted that in cases where terms were renamed or relabelled, the original labels were retained as a synonym. A summary of the revisions to date is presented in Table 3. The revisions are available in the current release of Mondo (https://github.com/monarch-initiative/mondo/releases), viewable on OLS (https://www.ebi.ac.uk/ols4/ontologies/mondo/classes/http%253A%252F%252Fpurl.obolibrary.org%252Fobo%252FMONDO_0005027), or available for download on GitHub (https://github.com/monarch-initiative/mondo/releases).

**Table 3:** Summary of revisions to Mondo epilepsy branch.

Type of revision	Count
Add new term	26
Obsoletion	10
Merge	4
Add or revise synonym	165
Revise xref	24
Revise superclass/reclassify term	202
Exclude superclass	69
Rename/relabel	10
Revise definition	55

Note that the addition of a previous label as a synonym in the cases of rename/relabel are reported separately from the ‘add or revise synonym’ revision type.

## High-level classifications of epilepsy in ILAE

The ILAE classifies epilepsy terminology based on four key areas: seizure types [which are represented in Human Phenotype Ontology (HPO) [15] and were considered out of scope for Mondo and the workshop], epilepsy types, epilepsy etiologies, and epilepsy syndromes. The outcomes on the reclassification of Mondo epilepsy disease terms are described below.

### Classification by epilepsy types

Four main epilepsy types are represented in ILAE, which include (i) focal epilepsy, (ii), generalized epilepsy, (iii) combined generalized and focal epilepsy, and (iv) unknown onset epilepsy.

**Figure 2:**
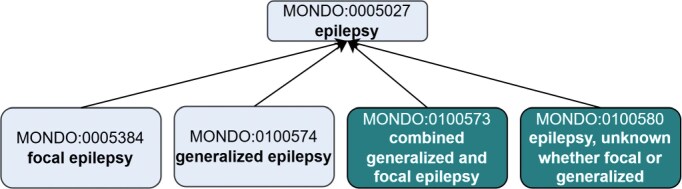
Creation of new ‘epilepsy type’ terms to align with the ILAE. Existing terms are depicted in lighter shading and new terms that were added as an outcome of the workshop are depicted in darker shading. The new terms were asserted as subclasses of MONDO:0005027 epilepsy. Note, this is just a partial representation of the classification, not all of the asserted subclasses of ‘epilepsy’ are depicted.

Certain classifications based on epilepsy types were not yet represented in Mondo. We introduced them by creating the following terms: (i) ‘combined generalized and focal epilepsy’ (MONDO:0100573) and (ii) ‘epilepsy, unknown whether focal or generalized’ (MONDO:0100580) (Fig. 2). SubClasses of these terms were added according to ILAE classification (details here: https://github.com/monarch-initiative/mondo/issues/8280).

#### How to best represent epilepsies where the etiology is unknown or may change over time?

Patients with rare diseases often go through a ‘diagnostic odyssey’. This refers to the often lengthy, frustrating journey patients and families face when seeking a diagnosis for rare epilepsies [16]. The time from seizure onset to genetic diagnosis has decreased with more widespread availability of genetic testing [17], but diagnosis can be delayed for many patients, even into adulthood [18–20]. The process often includes multiple specialist referrals (neurologists, epileptologists, and geneticists), numerous tests with inconclusive results, misdiagnoses, and ineffective treatments. These patients who have seizures are initially given an epilepsy diagnosis where the cause is not known and the onset is unknown. As more advanced and differentiating diagnostic procedures—such as genetic testing, genome or exome sequencing, EEGs, and MRIs—are performed, the whole process becomes increasingly refined, resulting in more specific and accurate diagnosis over time.

When annotating data from these types of patients, curators choose the most specific ontology term for the known epilepsy diagnosis at that given time. For example, we would use the broadest ontology term, epilepsy (MONDO:0005027), to represent the overall epilepsy diagnosis. Using this more non-specific ontology term indicates that a patient has epilepsy and may later be diagnosed with a more specific form of epilepsy, such as focal epilepsy. When patients receive a more specific diagnosis, a more specific term is used (e.g. focal epilepsy, MONDO:0005384). These more specific terms are represented in the ontology as subclasses (or ontological children) of the general term epilepsy.

Because of the hierarchical structure of Mondo (and ontologies in general), all annotations to specific terms inherently carry the meaning of their broader ontological parent terms. This allows annotations to remain interoperable and semantically valid across varying levels of data granularity. In practice, curators can annotate using high-level terms when only general information is available or select deeper, more granular terms when richer clinical or genomic data exist. This flexible structure ensures consistent data representation and supports both broad-scale analyses and precise phenotype–genotype correlations.

**Figure 3:**
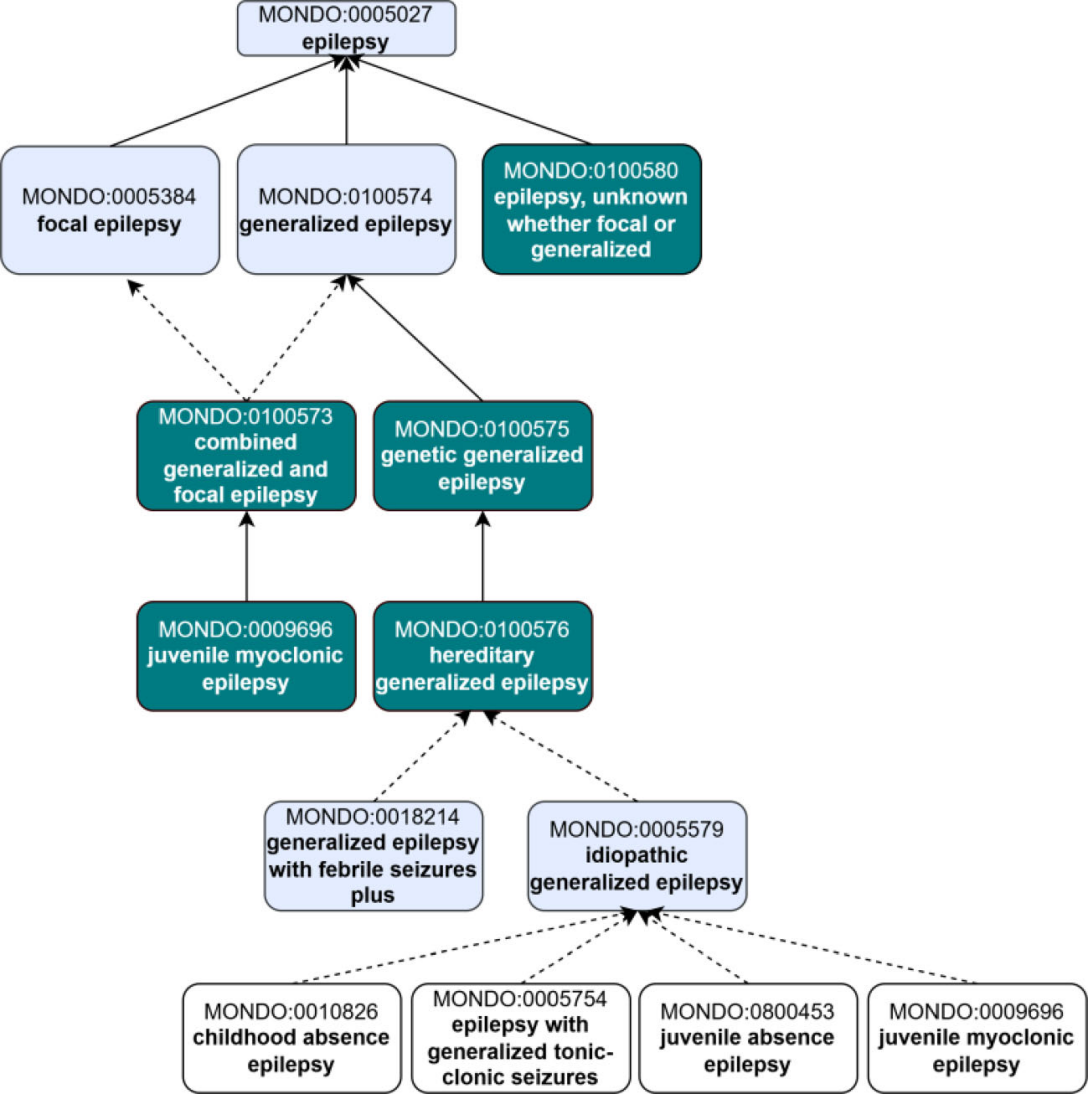
Revisions to the top-level epilepsy hierarchy in Mondo to introduce new ‘epilepsy types’ in alignment with the ILAE. Existing terms are depicted in lighter shading, and new terms that were added as an outcome of the workshop are depicted in darker shading. Terms that were reclassified are indicated in white (no shading). Dotted line indicates the classification is inferred.

The term ‘epilepsy, unknown whether focal or generalized’ (MONDO:0100580) is used when a patient is diagnosed with epilepsy, but the epilepsy type cannot be determined. This may occur due to insufficient clinical information, such as unavailable or inconclusive EEG, or unclear seizure semiology. For example, a patient may have experienced symmetrical tonic–clonic seizures with normal EEG findings, leaving the seizure onset—and thus the epilepsy type—uncertain. In some cases, the seizure type may also be unknown, although seizure and epilepsy types are not always concordant. This term can also apply when comprehensive diagnostic evaluation has been completed, all specific epilepsy types have been ruled out, and the epilepsy type remains unclear. While such a diagnosis is valid, it is often used with the understanding that a more specific classification may become possible as new clinical information emerges (Fig. 3).

#### Focal and generalized epilepsies

Additional subclasses of the epilepsy types were created or reclassified to align with the ILAE. We created a new term, ‘genetic generalized epilepsy’ (MONDO:0 100 575) to group epilepsy terms based on genetic etiology (Fig. 3).

The concept ‘idiopathic generalized epilepsy’ (a subtype of ‘generalized epilepsy’) is used by experts to represent four specific epilepsy diseases: ‘childhood absence epilepsy’ (MONDO:0010826), ‘epilepsy with generalized tonic-clonic seizures’ (MONDO:0005754), ‘juvenile absence epilepsy’ (MONDO:0800453), and 'juvenile myoclonic epilepsy’ (MONDO:0009696). An existing Mondo term, labelled ‘epilepsy, generalized idiopathic’ (MONDO:0005579), misrepresented the concept as a general epilepsy whose etiology was unknown. We updated the label of this term to ‘idiopathic generalized epilepsy’ to represent the terminology used by the experts, updated the definition and axioms (we removed the information about the etiology being unknown), and updated the classification accordingly (see GitHub issue: https://github.com/monarch-initiative/mondo/issues/8280). The term ‘idiopathic generalized epilepsy’ (MONDO:0005579) is inferred to be a subclass of ‘hereditary generalized epilepsy’ (MONDO:0100576), and groups the four epilepsy diseases as reported above (Fig. 3).

### Classification by epilepsy etiologies

ILAE classifies epilepsies based on etiologies: structural, genetic, infectious, metabolic, immune, and unknown etiology. We reviewed this classification in Mondo and updated the ontology accordingly.

(i) Structural epilepsy—(MONDO:0100035) no revisions were needed to the current classification in Mondo.
(ii) Genetic epilepsy—Currently in Mondo, we have a subclassification by ‘monogenic epilepsy’ (MONDO:0015653), i.e. the etiology is inherited and is due to variation(s) in a single gene. Experts recommended classifying genetic epilepsy in a more rigorous way beyond just ‘monogenic epilepsy’. We may expand ‘genetic epilepsy’ to not only include monogenic epilepsy, but also epilepsies secondary to copy number variants and epilepsies with a significant polygenic-driven risk in the future. In addition, ‘genetic epilepsy’ would also cover other genetic disruptions which are not inherited, e.g. de novo mutations. This requires further discussion amongst the Mondo curation team and will have implications for other genetic forms of diseases, so it will be revisited later, and changes will be made accordingly after further discussions.
(iii) Infectious epilepsy—The term ‘epilepsy due to infection’ is not currently included in Mondo. Upon review, we found that conditions grouped under ‘infectious epilepsy’ by the ILAE primarily represent infectious diseases in which seizures may occur as a symptom, rather than distinct types of epilepsy. Based on expert recommendations, we decided not to include an ‘infectious epilepsy’ classification in Mondo, as it does not reflect a discrete epilepsy entity but rather a set of underlying infectious conditions with seizure manifestations.
(iv) Metabolic epilepsy—(MONDO:0100033) no revisions were needed to the current classification in Mondo.
(v) Immune epilepsy—Experts recommended retaining the term ‘immune epilepsy’ (MONDO:0100028) and its subclass ‘antibody-mediated epilepsy’ (MONDO:0100029), as both are recognized in the ILAE classification. However, while ‘antibody-mediated epilepsy’ is represented in ILAE, the current Mondo subclasses—anti-NMDA receptor encephalitis (MONDO:0021081), steroid-responsive encephalopathy associated with thyroid disease (MONDO:0019385), and celiac disease, epilepsy and cerebral calcification syndrome (MONDO:0009187)—are not epilepsy types themselves, but rather distinct diseases in which seizures may occur. As a result, we removed these subclasses from under ‘antibody-mediated epilepsy’ in Mondo and added a comment to document the rationale for this change. Further action is being taken into consideration to merge ‘autoimmune epilepsy’ into ‘antibody-mediated epilepsy’, which is further described here: https://github.com/monarch-initiative/mondo/issues/9267.
(vi) Unknown epilepsy—We reviewed the epilepsy terms grouped under this classification in ILAE to ensure that they are classified correctly in Mondo. These terms include ‘Rasmussen syndrome’ (‘Rasmussen subacute encephalitis’ MONDO:0016019) and ‘febrile infection-related epilepsy syndrome’ (FIRES) (MONDO:0015584). These two diseases have additional classifications in Mondo: Rasmussen syndrome is currently classified under three classes: ‘postinfectious encephalitis’ (MONDO:0020068), ‘encephalitis’ (MONDO:0019956), and ‘epilepsy’ (MONDO:0005027). FIRES is classified as an ontological child of ‘childhood-onset epilepsy syndrome’ (MONDO:0020072). We decided to revisit the classification of these two diseases after we have implemented other changes. According to ontology best practices, terms such as ‘unknown’ etiology are rarely represented in ontologies. Following the best practices, we did not create a new classification for ‘unknown etiology’ in Mondo. Curators using Mondo for disease annotations should annotate to the next highest level term.

#### Note about ‘genetic epilepsy’

As more evidence is accumulating for the underlying genetic etiology of many rare epilepsies, it became apparent that the Mondo classification approach of genetic epilepsies should be revisited. We discussed the proposal to create a new ‘genetic epilepsy’ grouping class that groups hereditary and non-hereditary forms of the disease; the term ‘monogenic epilepsy’ would be created as a subclass of ‘hereditary epilepsy’. There was discussion about distinguishing hereditary forms of a disease from a sporadic (de novo) form, as many genetic epilepsies are sporadic rather than inherited. This was previously discussed in a specific example on this GitHub issue about tuberous sclerosis (https://github.com/monarch-initiative/mondo/issues/8074), where the variations causative to the disease can be inherited or appear de novo in the patient. In most cases, when the patient is able to reproduce, these sporadic variations could be passed on, and therefore the disease would be hereditary. Therefore discussions are ongoing to determine whether the distinction between ‘de novo’ and ‘inherited’ variation is necessary and if so, how we can best maintain this in the ontology. An open GitHub issue regarding genetic epilepsies that diagrams the proposed new approach and discusses the proposal in more detail is here (https://github.com/monarch-initiative/mondo/issues/8483), and public comments are welcomed. These changes will have broader implications on Mondo in general. If this new proposed pattern is accepted, we would apply this across other higher-level disease areas, such as cardiovascular or neurological.

In refining these classifications, it is essential to clarify key terminology. The term hereditary refers to traits or conditions that show familial segregation, typically implying a genetic component—though in some cases, the exact genetic basis may be unknown. In contrast, genetic is a broader term that encompasses various forms of genomic alteration, including monogenic, polygenic, chromosomal, mitochondrial, and even epigenetic mechanisms, where gene expression is modified without a change in the DNA sequence. In some cases, particularly when a genetic alteration occurs postnatally and is triggered by environmental or external factors, the term acquired may be more appropriate. For instance, while acquired channelopathies are theoretically possible, they are less commonly recognized or described in the literature compared to inherited forms.

This evolving framework aims to ensure that Mondo accurately captures both biological mechanisms and clinical realities, supporting more precise disease representation and downstream applications in diagnostics, research, and therapeutic discovery.

### Epilepsy syndromes

#### Definition of epilepsy syndrome

Both Mondo and ILAE include the term ‘epilepsy syndrome’ (MONDO:0015650), which was undefined in Mondo. An open question is whether ‘epilepsy syndrome’ represents a syndrome that includes an epilepsy feature, or in which the main feature is epilepsy. A new definition was proposed based on the definition from ILAE: ‘A characteristic cluster of clinical and EEG features, often supported by specific etiological findings (structural, genetic, metabolic, immune, and infectious).’ A survey of the workshop participants—whose expertise included clinicians, researchers, geneticists, caregivers/parents, and patient advocates, with some individuals holding multiple roles—revealed that 27% (4) agreed with the definition, 53% (8) were uncertain or partially agreed, and 20% (3) disagreed. Based on their feedback, a new definition was drafted: ‘An epilepsy syndrome is a characteristic cluster of clinical features and/or electrographic (EEG) findings that reflect underlying epileptic activity. It is often associated with a range of other health issues, including cognitive impairment, intellectual disability, physical gross motor and fine motor delays, speech and language deficits, and impacts to other bodily functions, etc., and may be supported by specific etiological findings—such as structural, genetic, metabolic, immune, or infectious causes or have an unknown etiology.’ A comment was included to note that a single individual may have multiple epilepsy syndromes and/or multiple etiologies, and the presentation can include a wide variety of symptoms. It is important to note, however, that the representation of syndromes in Mondo, including epilepsy syndrome, is still being discussed. Upcoming discussions will involve a wider group of experts, and consensus will be achieved through Ontaccord, our group’s Delphi-style ontology platform [21]. A ticket related to the definition of ‘epilepsy syndrome’ remains open on GitHub (https://github.com/monarch-initiative/mondo/issues/9270) for further comment and discussion.

##### Classification of syndromes as an ‘epilepsy syndrome’ and ‘non-syndromic epilepsy’

Mondo allows for multiple-classification, i.e. a term can have more than one superclass. A syndromic term that includes a non-syndromic epilepsy type should be classified as both an ‘epilepsy syndrome’ and the epilepsy type. For example, ‘variable-age onset focal epilepsy syndrome’ (MONDO:0800492) and ‘childhood-onset self-limited focal epilepsy syndrome’ (MONDO:0800496) should both be subclasses of ‘epilepsy syndrome’ (MONDO:0 015 650) and ‘focal epilepsy’ (MONDO:00 005 384).

##### Representation of age-specific epilepsy syndromes in Mondo

**Figure 4:**
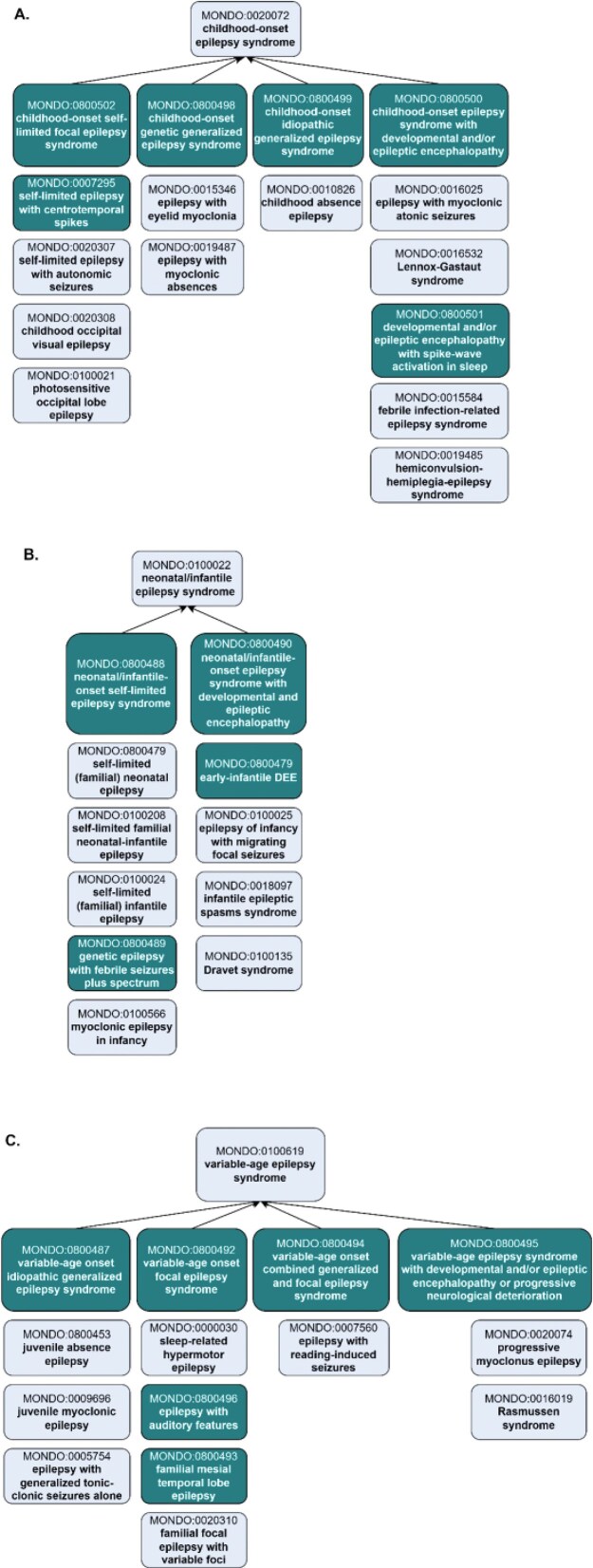
Representation of age-specific epilepsy syndromes in Mondo, revised to align with ILAE. (a) The ‘childhood-onset epilepsy syndrome’ was revised to reclassify terms and add new grouping classes and new concepts. (b) The ‘neonatal/infantile epilepsy syndrome’ and the (c) ‘variable age epilepsy syndrome’ branch include the addition of new classes and revisions to the existing classification. Subclasses are depicted vertically below the grouping classes. Existing terms are depicted with lighter shading and new terms that were added as an outcome of the workshop are depicted with darker shading. For simplicity, not all of the asserted subclasses are shown.

The ILAE includes classifications based on age of onset-specific subtypes of epilepsy syndrome: ‘childhood-onset epilepsy syndrome’ (MONDO:0020072) and ‘neonatal/infantile epilepsy syndrome’ (MONDO:0100022) and ‘variable age epilepsy syndrome’ (MONDO:0100619). ILAE includes additional grouping subclasses within each of these grouping classes, e.g. ‘ childhood-onset genetic generalized epilepsy syndrome’. These grouping classes were added to Mondo and aligned the hierarchy accordingly (Fig. 4).

##### Challenges with temporal disease progression

Ontologies often face challenges in accurately representing disease progression and the temporal dynamics of a patient’s clinical course. In particular, terms denoting age-specific onset can introduce ambiguity during disease annotation. For example, in the case of a patient diagnosed with *juvenile myoclonic epilepsy* (JME) (MONDO:0009696) at age 13, questions may arise regarding whether the ‘juvenile’ diagnosis remains appropriate when the patient is later assessed in adulthood. This highlights the critical importance of annotating data to the ontology term definition rather than relying solely on the term label. According to its definition, JME is ‘the most common hereditary idiopathic generalized epilepsy syndrome, characterized by adolescent-onset myoclonic jerks, generalized tonic-clonic seizures, and absence seizures in ∼30% of cases.’ Given that JME is defined by its characteristic age of onset rather than by the patient’s age at the time of diagnosis or follow-up, it is appropriate for the diagnosis to persist into adulthood.

##### Obsoletion of syndromic epilepsy grouping classes

Several grouping classes in Mondo (listed below) were no longer used in ILAE and were obsoleted accordingly.

- MONDO:0 020073 ‘adolescent-onset epilepsy syndrome’.
- MONDO:0100030 ‘adolescent/adult-onset epilepsy syndrome’.
- MONDO:0020627 ‘epileptic encephalopathy, infantile or early childhood.

The only age of onset classes are now ‘neonatal/infantile epilepsy syndrome’ (MONDO:0020070), ‘childhood-onset epilepsy syndrome’ (MONDO:0020072) and ‘variable age epilepsy syndrome’ (MONDO:0100619), which aligns with ILAE.

The potential merger of ‘infantile epilepsy syndrome’ (MONDO:0020071) and ‘neonatal epilepsy syndrome’ (MONDO:0020070) into a single entity, neonatal/infantile epilepsy syndrome (MONDO:0100022), was considered. However, it was determined that these represent clinically distinct syndromes with differing onset periods and diagnostic implications. As both terms are used independently in clinical practice, they have been retained as separate concepts within the ontology.

##### Reclassification of syndromic epilepsy terms

Moynahan syndrome (MONDO:0008755) was reclassified as a subclass of Mendelian disease and removed from the ‘epilepsy syndrome’ and ‘hyperpigmentation of the skin’ branches, as epilepsy and the skin abnormalities are components of the syndrome and are not the disease-defining features.

##### Addition of ‘myoclonic epilepsy’ grouping term

A new grouping class, ‘myoclonic epilepsy’ (MONDO:0100577) was added to group together 33 myoclonic epilepsy concepts, including infantile (MONDO:0011506), juvenile (MONDO:0009696), and adult forms (MONDO:0000160); progressive forms (MONDO:0020074) and specific subtypes (‘myoclonic epilepsy in non-progressive encephalopathies’) (MONDO:0019488) and ‘myoclonic epilepsy, Hartung type’ (MONDO:0008041).

#### Developmental and epileptic encephalopathy

Over the years, Mondo has made iterative changes to the ‘developmental and epileptic encephalopathy’ branch, including renaming of the term to ‘developmental and epileptic encephalopathy’ from ‘early infantile epileptic encephalopathy’. Upon recent review, it was pointed out that there are non-hereditary forms of DEE, but the current DEE class corresponded to an OMIM phenotypic series (https://www.omim.org/phenotypicSeries/PS308350), which is always considered genetic. Therefore, MONDO:0100062 was renamed to ‘genetic developmental and epileptic encephalopathy’, and we created a generic DEE class as a superclass of the genetic DEE class. MONDO:0100062 ‘genetic developmental and epileptic encephalopathy’ was labelled as genetic instead of hereditary account for cases that are caused by de novo variations and not inherited. There sometimes may be cases that are ‘presumed genetic’ where a clinical judgment is based on current evidence and knowledge, acknowledging that genetic mechanisms are highly likely or there may be variations of unknown significance but not yet proven in that individual [22].

**Figure 5:**
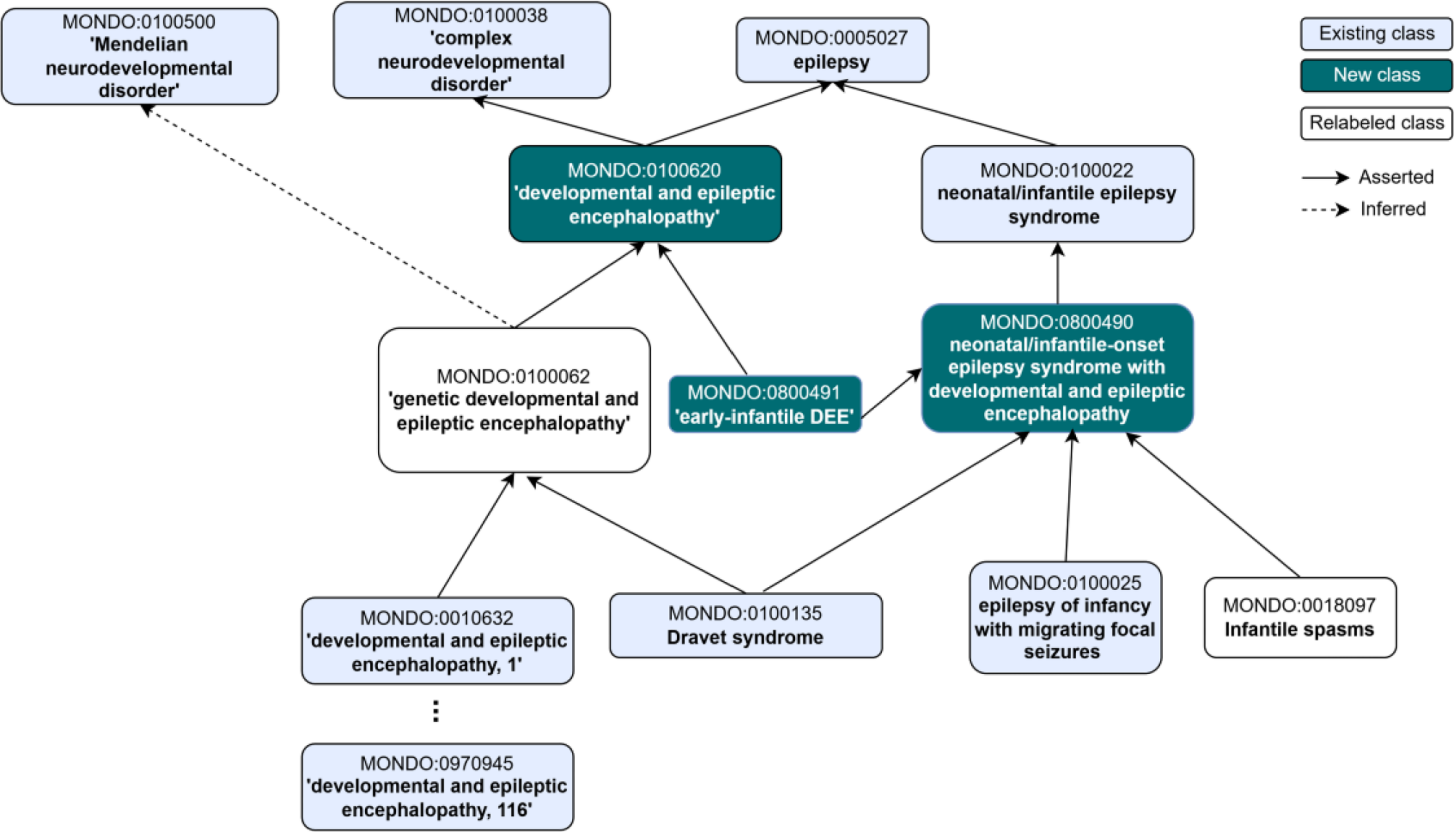
Revised representation of ‘developmental and epileptic encephalopathy’. Major revisions are still underway, and currently we are considering the addition of the generic ‘developmental and epileptic encephalopathy’ and relabelling of MONDO:0100062 to ‘hereditary developmental and epileptic encephalopathy’. Additionally, DEE is asserted as a subclass of epilepsy (whereas previously it was a subclass of ‘complex neurodevelopmental disorder’, only without any relationship to epilepsy). The new term ‘early-infantile DEE’ was added as part of the alignment with ILAE, and the cross-references to external ontologies and terminologies were revised. Another major change in this branch was the renaming of ‘West syndrome’ to ‘Infantile Spasms’, which is the currently accepted label for this term.

In past discussions (https://github.com/monarch-initiative/mondo/issues/1835) and recent discussions, questions came up about how to best classify the term ‘Dravet syndrome’. OMIM (https://omim.org/entry/607208) treats Dravet syndrome as equivalent to ‘developmental and epileptic encephalopathy 6A’, which is caused by variations in the SCN1A gene. However, not all cases of Dravet syndrome are caused by SCN1A [23–25], therefore we needed to classify Dravet syndrome as a separate entity from MONDO:0100079 ‘developmental and epileptic encephalopathy, 6A’, diverging from OMIM’s classification. Evidence suggests that SCN1A variants are found in 80% of Dravet cases [26]. These revisions were confirmed with members of the ClinGen Epilepsy Gene Curation Expert Panel (https://clinicalgenome.org/affiliation/40005/) in a separate follow-up meeting. The reclassification is depicted in Fig. 5 below and the discussion can be followed on our GitHub issue tracker (https://github.com/monarch-initiative/mondo/issues/8274).

**Figure 6:**
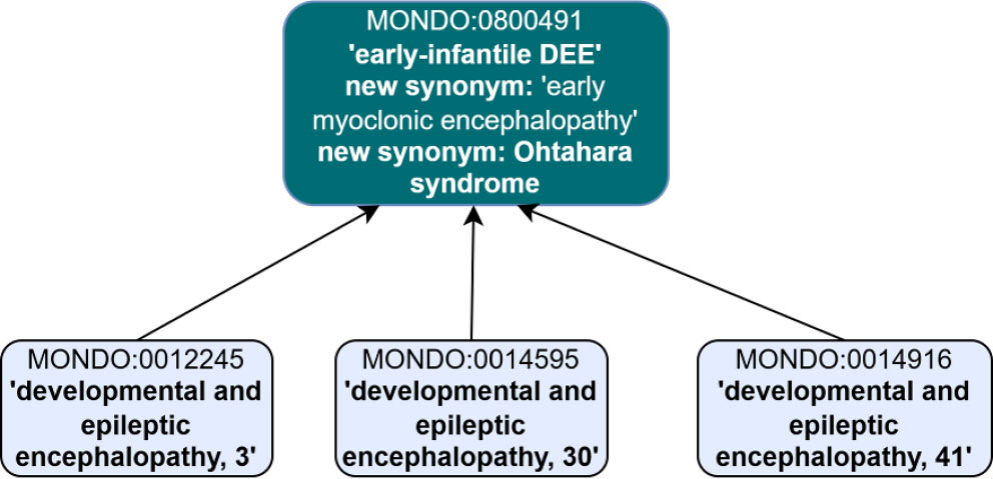
Revisions to MONDO:0800491 ‘early-infantile syndrome’ included revised synonyms and subclasses. ‘Early myoclonic encephalopathy’ is proposed to be merged into ‘early-infantile DEE’ and ‘Ohtahara syndrome’ is proposed to be a new synonym. The subclasses were revised, and these three DEE subtypes remain as subclasses.

Further revisions were made to ‘early-infantile DEE’ (MONDO:0800491). Per the definition of ‘early-infantile DEE’ in ILAE, which states ‘This syndrome encompasses the previously defined syndromes of Ohtahara syndrome and early myoclonic encephalopathy’, we proposed merging ‘early myoclonic encephalopathy’ (MONDO:0016022) into ‘early-infantile DEE’ and moving the synonym ‘Ohtahara syndrome’ to be an exact synonym of ‘early-infantile DEE’ (instead of a synonym of DEE itself) (Fig. 6). Additionally, two subclasses were excluded as subclasses of ‘early-infantile DEE’: ‘familial infantile myoclonic epilepsy’ (MONDO:0011506) and ‘myoclonic epilepsy, Hartung type’ (MONDO:0008041). These two terms are now subclasses of the new ‘myoclonic epilepsy’ (MONDO:0100577) class.

### Disease naming in Mondo

The way diseases—particularly epilepsy syndromes—are named carries profound implications for clinical care, research, and access to services. Names shape how healthcare providers interpret conditions, influence diagnostic clarity, and directly affect whether patients receive insurance-covered therapies. Many established epilepsy syndromes, such as Lennox–Gastaut Syndrome (LGS) or Infantile Spasms (IS), were defined through decades of clinical observation before the genomic era, and these names are still recognized by care systems and payors. Abruptly shifting to etiology-only naming risks leaving patients behind, as support systems often lag in adopting new terminology. To minimize harm, it is essential to maintain historical syndrome names alongside newer genetic diagnoses—e.g. identifying a condition as ‘LGS secondary to SCN3A mutations.’ Mondo aims to support this need by reflecting all community preferences for disease naming without arbitrating official names. For each disease, Mondo provides all relevant names as synonyms, tracks their provenance, and allows community-specific preferred names. This inclusive and flexible approach ensures Mondo serves a wide range of communities, supporting accurate communication, continuity of care, and access to services for families navigating complex neurological disorders.

### Disease vs. phenotype

A big question for Mondo has always been what is considered a disease and what is considered a phenotype. We work closely with and aim to align with the HPO to ensure minimal overlap between the two ontologies, but there are some cases where terms appear in both ontologies. For example, diabetes mellitus is a disease (MONDO:0005015) but sometimes it can be a feature of a disease, in which case it could be considered a phenotype (HP:0000819). We reviewed classes in Mondo for their suitability as a disease vs. a phenotype. We concluded that the two terms, ‘status epilepticus’ (MONDO:0002125) and ‘epilepsia partialis continua’ (MONDO:0006748) are both phenotypes that occur in epilepsy disorders. These two terms were obsoleted and the corresponding HPO terms were recommended as alternatives.

The term electroclinical syndrome is a legacy term that was once included in ILAE, but is no longer used. Upon discussion, it was noted that electroclinical syndrome refers to phenotypic features and therefore is not suitable for Mondo, and it was obsoleted accordingly. Absence epilepsy is also considered a phenotypic feature and not a disease entity, and therefore is not suitable for inclusion in Mondo and was obsoleted accordingly (see: https://github.com/monarch-initiative/mondo/issues/8468).

### Rare Disease Cures Accelerator—Data and Analytics Platform

**Figure 7:**
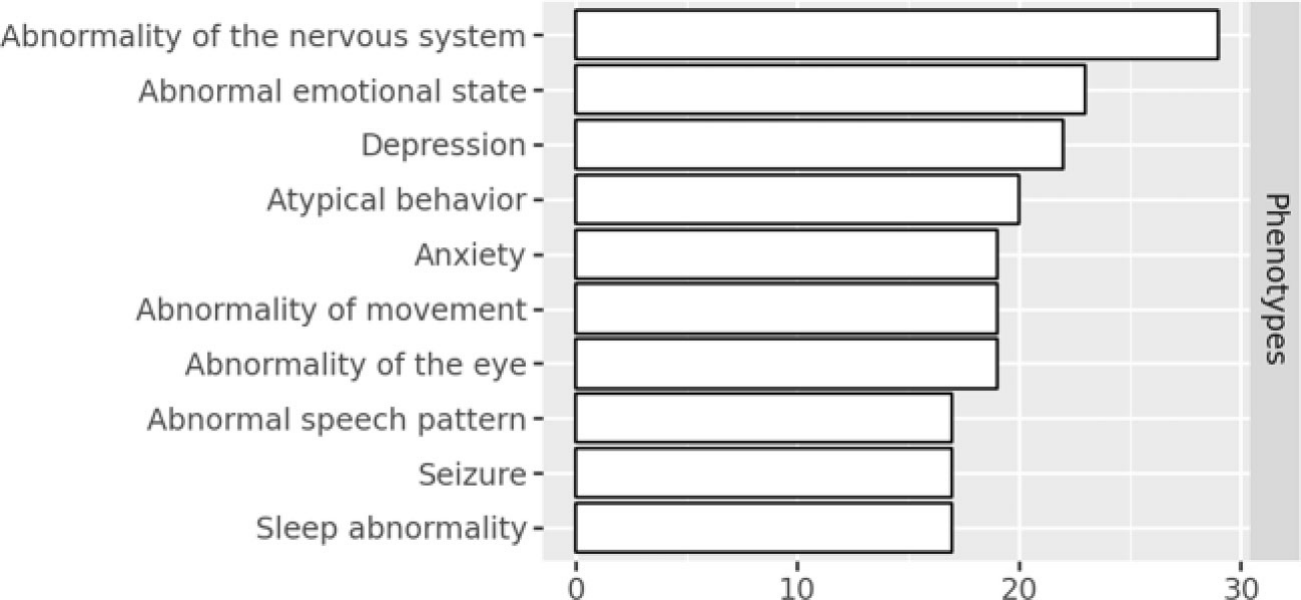
The top phenotypes that are characteristic in rare epilepsy datasets in the C-Path KG.

A major driver for hosting this workshop series was the need for standardization of epilepsy datasets in the Rare Disease Cures Accelerator-Data and Analytics Platform (RDCA-DAP: https://portal.rdca.c-path.org/). The RDCA-DAP, led by the Critical Path Institute (C-Path) in partnership with the U.S. Food and Drug Administration (FDA), provides a centralized data repository that integrates patient-level data from various sources, including clinical trials, natural history studies, patient registries, and real-world data such as electronic health records. It supports the acceleration of drug development by promoting the standardization and open sharing of rare disease data, including rare epilepsy and seizure-related disorder datasets. The RDCA-DAP currently houses a collection of 13 epilepsy/seizure-related disorder datasets, such as the Rare Epilepsy Network (REN) (www.rareepilepsynetwork.org/), which covers a wide range of epilepsy-related concepts and comorbidities, as well as disease-specific datasets covering disorders such as Kleefstra syndrome (MONDO:0012455), ‘CACNA1A-related complex neurodevelopmental disorder’ (MONDO:0100254), and ‘tuberous sclerosis’ (MONDO:0001734). These datasets include data from patient registries, observational studies, clinical trials, and natural history studies. Mondo is leveraged within RDCA-DAP to harmonize disease terminology and enable the integration of disease information across varied datasets. Select data from the RDCA-DAP is integrated into a knowledge graph that utilizes the Monarch Initiative’s Knowledge Graph (KG) [27]. A number of epilepsy disease concepts, including new concepts that were recently added to Mondo are represented in the data (Table 4). The KG can be queried to reveal connections between disease concepts and other features, such as disease to phenotype relationships as illustrated in Fig. 7.

**Table 4:** Disease concepts represented in the C-Path KG.

Disease name	Count
Absence epilepsy	2
Childhood absence epilepsy	2
Childhood electroclinical syndrome	2
Childhood-onset epilepsy syndrome	2
Electroclinical syndrome	2
Epilepsy syndrome	2
Generalized epilepsy	2
Genetic generalized epilepsy	2
Hereditary generalized epilepsy	2
Idiopathic generalized epilepsy	2
Developmental and epileptic encephalopathy	1
Early myoclonic encephalopathy	1
Lennox-Gastaut syndrome	1
Neonatal/infantile epilepsy syndrome	1
Neonatal period electroclinical syndrome	1

## Discussion

Mondo enables consistent representation of diseases such as epilepsy and related seizure disorders across clinical, genetic, and phenotypic datasets. This standardized structure supports data interoperability, facilitates cross-species comparisons, and enables computational reasoning to uncover disease-gene relationships and phenotypic similarities, advancing research and precision medicine for complex and rare diseases.

### Use cases

Mondo is used in clinical use cases, for epilepsy research, and biomedical databases to standardize disease annotations, enabling consistent classification and integration of epilepsy-related data across diverse sources. Specific use cases and forward-looking opportunities are described below.

#### Computational drug repurposing

Mondo can be leveraged to support computational approaches to drug repurposing, particularly for rare diseases where high-quality, standardized disease information is critical. One of the key issues facing diseases that affect a small number of the patient population is the lack of availability of treatments. This is most often because the small prevalence in the population makes the large investment into the development of pharmaceutical or other therapeutic interventions difficult to justify; especially as safety and efficacy trials for novel drugs take many years and are incredibly expensive [28]. One promising alternative to developing new drugs is the repurposing of existing treatments—using medications already approved and deemed safe for one condition to treat another. Given the vast number of FDA-approved drugs, identifying viable repurposing opportunities requires substantial effort, making computational methods essential for prioritizing candidates. These methods rely on highly accurate and specific disease annotations to draw meaningful connections between conditions and potential therapies. The refined disease classifications and annotations developed through this work can support future efforts to identify clusters of patients with rare epilepsies who may benefit from approved treatments, accelerating the path to care. This approach aligns with ongoing initiatives such as REMEDi4ALL (https://remedi4all.org/) and Every Cure (https://everycure.org/), which aim to match existing drugs to new disease indications, including rare neurological conditions.

#### Data for the Common Good

Data for the Common Good (https://commons.cri.uchicago.edu/) is a collaborative initiative based at the University of Chicago that harnesses the power of data to drive scientific discovery and improve human health. Working with global partners, D4CG develops data commons—centralized, standardized repositories of health data—that support research across rare diseases, cancer, and social determinants of health. Building on its success in pediatric cancer, D4CG is expanding into other domains, including pediatric monogenic epilepsies. These efforts aim to create comprehensive data commons that integrate clinical, genomic, and social data, standardized using community-recognized terminologies. As part of this effort, D4CG may incorporate the Mondo Disease Ontology to support more consistent and interoperable disease classification.

One specific use case involves refining the representation of genetic epilepsies within Mondo. Rather than limiting coverage to the current term for monogenic epilepsy (MONDO:0015653), D4CG seeks to include broader classifications such as polygenic epilepsy—where complex copy number variants or combinations of variants across multiple genes contribute to disease—as well as cases caused by de novo mutations. To support these needs, the D4CG and Mondo teams will collaborate to discuss future efforts to expand the ontology to reflect the full spectrum of genetic epilepsy, enabling more precise data capture and improved research utility across D4CG’s platforms.

#### Clinical genome resource

Clinical Genome Resource (ClinGen) (https://clinicalgenome.org/) uses Mondo to standardize disease terminology across its variant curation efforts, ensuring consistency in how genetic variants are linked to clinically recognized conditions. This harmonization enables more accurate variant interpretation and facilitates data sharing across research and clinical communities. Members of the Epilepsy Gene Curation Expert Panel (https://clinicalgenome.org/affiliation/40005/) demonstrated the gene-disease validity for DNM1 in DEEs, where there was a definitive association for the autosomal dominant form (MONDO:0014598 ‘developmental and epileptic encephalopathy, 31A’) and a moderate association for the autosomal recessive form (MONDO:0957248 ‘developmental and epileptic encephalopathy, 31B’) (more details available here: https://search.clinicalgenome.org/kb/genes/HGNC:2972).

The ClinGen expert curators have been instrumental in providing new recommended disease terminology to Mondo, such as the addition of the new grouping term ‘DNM1-encephalopathy and neurodevelopmental disorder’ (MONDO:0700339) and assisting with defining new disease nomenclature, such as the revision of the label ‘early infantile epileptic encephalopathy’ to ‘developmental and epileptic encephalopathy’ in 2021. Mondo and ClinGen will continue to collaborate closely to ensure that disease terminology remains current and accurately classified, supporting high-quality variant interpretation and alignment with evolving clinical and research standards.

#### Utilizing ontology-based mapping to extract longitudinal phenotypes from EMR data in childhood genetic epilepsies

Genetic factors are increasingly implicated in childhood epilepsies and the disease course in childhood epilepsies is not static over time. This study [29] leveraged electronic medical record (EMR) data from 658 individuals with known or presumed genetic epilepsies enrolled at the Children’s Hospital of Philadelphia. Using a custom dictionary, clinical diagnoses and problems from the EMR were mapped to HPO terms, including higher-level ancestral terms through propagation. These phenotype data were binned into 100 discrete 3-month intervals from birth to age 25. Genotype–phenotype associations were then identified by comparing the frequency of HPO terms over time across 36 genetic etiologies seen in at least two individuals.

The study identified 528 HPO-mapped phenotypic features across 286 085 time-stamped entries, demonstrating that clinical features like febrile seizures and IS had time-dependent distributions consistent with known natural histories. Significant gene–phenotype associations emerged, including ‘Status epilepticus’ with *SCN1A*, ‘Severe intellectual disability’ with *PURA*, and ‘Infantile spasms’ with *STXBP1*. These findings highlight the importance of integrating longitudinal genotype–phenotype data into disease classification frameworks like the Mondo Disease Ontology, to improve the representation of dynamic, genetically driven epilepsies.

#### Applying disease ontologies in epilepsy precision care

With the increasing recognition of genetic etiologies in epilepsy and the growing number of precision medicine trials, there is an urgent need to establish specialized clinical services for diagnostic clarification and individualized management. Current clinical coding systems such as the International Classification of Diseases, 10th Revision (ICD-10), are optimized for common conditions but remain insufficient for the nuanced categorization of rare genetic epilepsies; at the time of writing, ICD-10 codes exist for only six monogenic epilepsy syndromes (Dravet syndrome [G40.83], CDKL5 deficiency disorder [G40.42], Rett syndrome [F84.2], SLC2A1-related disorder [E74.810], KCNQ2-related epilepsies[G40.84], and tuberous sclerosis [Q85.1]). In contrast, the Mondo Disease Ontology offers a disease-centred, hierarchically structured vocabulary comprehensively capturing the spectrum of rare epilepsies, with 351 epilepsy-related terms currently represented.

Workshop participants with experience in establishing and running clinical genetics services underscored Mondo’s potential as a standardized backbone for phenotype documentation in centres of excellence, multidisciplinary case conferences, and genetics clinics. Integration of Mondo terms into intake forms, diagnostic reports, and internal documentation would facilitate phenotype-driven data retrieval and cross-case comparisons. Critically, Mondo also enables interoperability with major genomic resources, including ClinVar and ClinGen, allowing seamless annotation of genetic variants with phenotype-associated meta information (gene-disease validity, mode of inheritance, etc.). Embedding Mondo as an electronic health record structured element would support the development of rare disease-specific decision support tools, automated alerts, and precision trial matching pipelines. Mondo captures age-dependent epilepsy syndromes defined by ILAE [30]. Consequently, it can be implemented both for pediatric and adult genetic epilepsy services and care. Alignment of Mondo with the ILAE Classification of Epilepsy Syndromes will further promote and incentivise the adoption of Mondo in clinical settings.

### Implications for patients and clinical care

Accurate classification of epilepsy-related disorders in Mondo is essential for capturing the full complexity of these conditions beyond just seizure symptoms. Referring to disorders by name alone, without recognizing their broader clinical impact, limits the effectiveness of care and overlooks the daily challenges faced by individuals and their families. A more precise and comprehensive understanding of each disorder will support systemic improvements in clinical management, enabling tailored interventions and care planning. For parents and caregivers, this clarity can help anticipate not only seizures but also associated comorbidities, allowing for proactive safety measures and more informed decision-making throughout the patient’s life.

## Future work

A few tickets which require further discussion and community consensus remain open in our GitHub repo. This will be addressed over time and in collaboration with subject matter experts and patient advocates.

### Classification of syndromes with multiple features

A longstanding question in Mondo is how to classify syndromic diseases in general—this applies to syndromic epilepsies and other syndromic diseases in other branches. We discussed the specific use case for MONDO:0008045 ‘spinal muscular atrophy-progressive myoclonic epilepsy syndrome’ (see https://github.com/monarch-initiative/mondo/issues/5655). The question is if this should be a subclass of ‘syndromic disease’ or a subclass of both ‘spinal muscular atrophy’ and ‘myoclonic epilepsy’. The preferred classification approach seems use case dependent and may vary depending on specific users and applications. The syndromic epilepsy definition requires a deeper discussion and remains an open topic. This is an ongoing discussion more generally amongst the Mondo curation team and we hope to come to a consensus on how to best model syndromic diseases in the future.

## Conclusion

Mondo’s continued improvement of structured representation of epilepsy enables better research, particularly in identifying genetic, exposure, and treatment-related factors, while also supporting data mining and analysis. Ultimately, Mondo enhances both clinical decision-making and scientific progress, contributing to improved patient outcomes and more efficient development of therapies. As the scientific and clinical landscape and understanding of epilepsy evolves, the Mondo team will continue to collaborate with external subject matter experts and consult the latest guidelines available from the ILAE, to ensure the representation of these concepts in Mondo stay scientifically accurate and up-to-date.

Open tickets for the epilepsy branch can be viewed here: https://github.com/monarch-initiative/mondo/issues?q=is%3Aissue%20state%3Aopen%20label%3Aepilepsy. Feedback and discussion is always welcome. The ontology will continue to evolve, and updates will be based on the latest scientific understanding and classifications. The team is committed to iterative improvements and keeping the community engaged in the process.

## Data Availability

The updated epilepsy classifications are publicly available in the latest Mondo release via GitHub (https://github.com/monarch-initiative/mondo/releases) and the Ontology Lookup Service (OLS).
